# Dentate line invasion as a predictive factor of poor distant relapse-free survival in locally advanced lower rectal cancer with anal sphincter involvement

**DOI:** 10.1186/s12885-022-10299-8

**Published:** 2022-11-19

**Authors:** Maxiaowei Song, Hongzhi Wang, Lin Wang, Shuai Li, Yangzi Zhang, Jianhao Geng, Xianggao Zhu, Yongheng Li, Yong Cai, Weihu Wang

**Affiliations:** 1 Key Laboratory of Carcinogenesis and Translational Research (Ministry of Education/Beijing), Department of Radiation Oncology, Peking University Cancer Hospital and Institute, Beijing, 100142 People’s Republic of China; 2 Key Laboratory of Carcinogenesis and Translational Research (Ministry of Education/Beijing), Department 3 of Gastrointestinal Surgery, Peking University Cancer Hospital and Institute, Beijing, 100142 People’s Republic of China

**Keywords:** Dentate line, Survival analysis, Failure patterns, Lower rectal cancer, Prognostic factor

## Abstract

**Background:**

While an important surgical landmark of the dentate line has been established for locally advanced lower rectal cancer (LALRC), the prognostic significance of dentate line invasion (DLI) has not been well defined. This study aimed to explore the impact of DLI on prognosis in LALRC patients with anal sphincter involvement after neoadjuvant chemoradiotherapy followed by surgery.

**Methods:**

We analyzed 210 LALRC patients and classified them into DLI group (*n* = 45) or non-DLI group (*n* = 165). The exact role of DLI in survival and failure patterns was assessed before and after propensity-score matching(PSM). Finally, 50 patients were matched.

**Results:**

Before matching, patients in the DLI group had poorer 5-year distant relapse-free survival (DRFS) (*P* < 0.001), disease-free survival (DFS) (*P* < 0.001), and overall survival (OS) (*P* = 0.022) than those in the non-DLI group, with the exception of local recurrence-free survival (LRFS) (*P* = 0.114). After PSM, the 5-year DRFS, DFS, OS, and LRFS were 51.7% vs. 79.8%(*P* = 0.026), 51.7% vs. 79.8%(*P* = 0.029), 71.6% vs. 85.4%(*P* = 0.126), and 85.7% vs. 92.0%(*P* = 0.253), respectively, between the two groups. DLI was also an independent prognostic factor for poor DRFS with (Hazard ratio [HR] 3.843, *P* = 0.020) or without matching (HR 2.567, *P* = 0.001). The DLI group exhibited a higher rate of distant metastasis before (44.4% vs. 19.4%, *P* < 0.001) and after matching (48.0% vs. 20.0%, *P* = 0.037) and similar rates of locoregional recurrence before (13.3% vs.7.9%, *P* = 0.729) and after matching (16.0% vs.12.0%, *P* = 1.000).

**Conclusions:**

DLI may portend worse DRFS and distant metastasis in LALRC patients with anal sphincter involvement, and this may be an important variable to guide clinicians.

**Supplementary Information:**

The online version contains supplementary material available at 10.1186/s12885-022-10299-8.

## Background

Neoadjuvant chemoradiotherapy (NCRT) followed by curative total mesorectal excision (TME) is a standard treatment for patients with stage II and III rectal cancer because local recurrence rates of 15% to 45% have dramatically decreased and survival has improved [1–3]. Although the survival of patients with locally advanced lower rectal cancer (LALRC) has improved, it is not to the same degree as that observed for mid- and upper-rectal cancer [4–6]. This is partly attributed to the more challenging surgery related to the narrow distal pelvis. And current studies also found that LALRC tends to metastasize to more distant lymph nodes (e.g., para-aortic, aortic bifurcation, and inguinal lymph nodes) and has a higher incidence of distant organ metastases [7, 8]. All these phenomena raise the possibility that LALRC may possess a distinct clinical behavior and require more closer follow-up. Moreover, intensive treatment might also significantly improve outcomes according to some of the clinical trials [9–11].

Pelvic magnetic resonance imaging (MRI) is the most accurate test to define locoregional clinical staging especially for LALRC and has wide application for evaluation or prediction tumor response [12–15]. LALRC with anal sphincter involvement refers to cases on MRI in which the lower edge of tumors directly invade the internal or external anal sphincter, which belongs to the surgical anal canal. The surgical anal canal comprises two parts: an endodermal portion above the dentate line and an ectodermal portion below. These segments have different innervations, vasculatures, and lymphatics. Theoretically, the clinical behavior of tumors above and below the dentate line should differ with respect to different sites of origin. In clinical practice, there is moderate evidence suggesting the above mechanism; however, there are few studies. Hamano et al. suggested that LALRC patients who have dentate line invasion (DLI) present a higher rate of inguinal lymph node (ILN) metastasis and poorer prognosis than those of patients without DLI [16]. Our previous results also revealed that DLI in LALRC patients was a pre-treatment factor associated with decreased 3-year disease-free survival (DFS) [17]. Although the above retrospective studies preliminarily confirmed the prognostic significance of DLI, its impact on local recurrences or distant metastases have seldom been systematically described or considered. More importantly, no prospective randomized controlled study has compared the prognosis of patients for whom the lower edge of tumors have DLI and those without DLI (non-DLI). Thus, the potential risks of DLI are ignored, and therapeutic or follow-up decisions are empirically made according to institutional routines rather than based on objective parameters.

Considering the potential risks of DLI, we conducted a real-world cohort study both before and after propensity-score matching (PSM) to assess the exact role of DLI in survival and failure patterns in LALRC patients with anal sphincter involvement after NCRT followed by TME.

## Methods

### Patients

The clinical data of 406 LALRC patients with internal or external anal sphincter involvement who had undergone NCRT from September 2010 to March 2020 were retrieved from a single institutional database and retrospectively reviewed. This study was conducted in accordance with the Declaration of Helsinki and was approved by the Ethics Committee of our hospital. The requirement for informed patient consent was waived owing to the retrospective nature of the study.

The inclusion criteria were: 1) histologically confirmed rectal adenocarcinoma with biopsy sample, 2) clinical stage T3 to T4 or any stage T and N + tumors without distant metastasis (based on the 7th edition of the American Joint Committee on Cancer), 3) an Eastern Cooperative Oncology Group (ECOG) performance status of 0–1 without any severe complications, 4) tumors invading the internal and/or external anal sphincter determined by pre-treatment MRI examination, 5) the lower edge of tumors had been assessed by pre-treatment endoscopy [18], and 6) patients who had undergone neoadjuvant long-course chemoradiation followed by TME. The exclusion criteria were: 1) occurrence of distant failure before surgery, 2) unsuitability for radical surgery by clinical examination and imaging, 3) history of pelvic radiation, 4) secondary malignancies, 5) incomplete clinical or pathological data, 6) NCRT or surgery in another hospital, and 7) previous recurrent rectal cancer.

We defined “dentate line invasion” as the lower edge of tumors involving or crossing the dentate line by colonoscopy [19]. Finally, 210 patients were included and classified into two groups according to whether the lower edge of tumors had DLI or not (DLI group and non-DLI group, respectively) as determined by pre-treatment endoscopy. In the endoscopic examination, the DLI were inspected using *en face* and retroflexed with white light and narrow band imaging [20].

### Treatment and follow-up

All patients received simultaneous integrated boost intensity-modulated radiation therapy. The definition and delineation of the clinical target volume (CTV) recommendations were in accordance with protocols described in previous reports [17, 21, 22]. A total dose of 50.6 Gy (2.3 Gy/fraction) and 41.8 Gy (1.9 Gy/fraction) both in 22 fractions were prescribed to the planning gross tumor volume and the planning target volume, respectively [22]. Concurrent chemotherapy consisted of capecitabine (825 mg/m^2^ orally twice daily, 5 days/week) with or without oxaliplatin (50 mg/m^2^ intravenous infusion once per week). Induction chemotherapy with 2–3 cycles of CAPEOX and consolidation chemotherapy including 2–4 cycles of CAPEOX or 1–2 cycles of capecitabine were also permitted. The time interval between NCRT and surgery was 5–12 weeks, except for patients who underwent consolidation chemotherapy. Adjuvant chemotherapy was recommended 4–6 weeks after TME using 4–6 months of CAPEOX/mFOLFOX6 (the modified leucovorin/fluorouracil/oxaliplatin regimen) or capecitabine. The protocol and time interval of the follow-up visits were consistent with our previous studies [21].

Additionally, follow-up was completed in the outpatient clinic during follow-up visits to record any readmissions or complications(≤ 12 months after surgery) after initial hospital discharge. The severity of postoperative complications was graded according to the Clavien–Dindo classification system [23].

### Study end points and statistics

In our previous study, DLI in LALRC patients was a pre-treatment factor associated with poor 3-year DFS, but not with local recurrence-free survival (LRFS); moreover, distant failure is more common than local recurrence [17]. In addition, systemic relapses remain a major problem in locally advanced rectal cancer [24]. Therefore, we chose distant relapse-free survival (DRFS) as the primary endpoint in this study. The secondary endpoints were DFS, overall survival (OS), LRFS, and failure patterns between the two groups. All events were calculated from the first day of NCRT until the first date of the event, death from any cause, or last follow-up. The locoregional recurrence sites were classified into four anatomical categories based on pelvic MRI or Computed Tomography(CT) including anterior (prostate, seminal vesicles, bladder, urethra, vagina, cervix, and uterus), posterior (presacral fascia and sacrum), lateral (ureter, hypogastric plexuses, internal iliac vessels, obturator neurovascular bundle or muscle, sciatic notch, and nerve roots), inferior (levator ani muscle and external anal sphincter) [25, 26], and anastomotic sites [21].

Differences between ratios were analyzed using the χ^2^ or Fisher’s exact test, as appropriate. The independent-samples *t-*test or Mann–Whitney *U* test were used to analyze continuous variables. PSM, an algorithm of the nearest neighbor and 1:1 matching with a caliper of 0.05, was used to balance the distribution of clinicopathologic variables between the two groups and to evaluate the exact role of DLI. A logistic regression model was used to calculate the propensity scores, and this took covariates such as sex, age, ECOG performance status, baseline serum carcinoembryonic antigen (CEA) level, tumor histologic grade, clinical T and N stages (cT and cN), preoperative serum CEA level, time interval between NCRT and surgery, type of surgery, surgical technique (laparoscopic and open surgery), operation duration, resection margin, perineural and lymphovascular invasion, yp T and yp N stages, treatment covariates (induction chemotherapy, concurrent chemotherapy, consolidation chemotherapy, adjuvant chemotherapy) and postoperative complications into account. Patients with one or more missing values for any of the variables considered were excluded from the matched analysis. Survival analyses were estimated using the Kaplan–Meier method and compared between groups using the log-rank test. Clinicopathological variables were entered into a Cox proportional hazard multivariate model and analyzed for effects on survival. Forest plots of the hazard ratios (HRs) of different subgroups were used to describe the stratified prognosis value of DLI.

Statistical analyses were performed using the SPSS 23.0 software package (IBM, Armonk, NY, United States) and the R software (version 3.6.1; http://www.r-project.org/). *P* values < 0.05 were considered statistically significant.

## Results

Among the 210 patients, 45 and 165 were in the DLI and non-DLI groups, respectively. The clinicopathological characteristics, treatment modalities and details of the operation of the two groups are shown in Table 1. The cT stage (*P* = 0.044),preoperative serum CEA levels (*P* = 0.027), type of surgery (*P* = 0.016) and postoperative complications (*P* = 0.008) were significantly different between the two groups before matching. Postoperative complications developed in 60 (28.6%) of the 210 patients. The anastomotic leakage (AL) rate in patients subjected to Low/ultralow anterior resection procedure/ Transanal total mesorectal excision/ Intersphincteric resection was 18.5% (5/27). Perineal wound complications developed in 37 (20.3%) of the 182 patients. The rate of small-bowel obstruction was 4.8% (10/210). Six patients (2.9%) developed postoperative bleeding. Four patients(1.9%) presented urinary complications.

**Table 1:** Baseline characteristics

Demographic variable	Before matching			After matching		
	Non-DLI group (*n* = 165) n (%)	DLI group (*n* = 45) n (%)	*P*	Non-DLI group (*n* = 25) n (%)	DLI group (*n* = 25) n (%)	*P*
**Sex**			0.622			0.765
Male	102(61.8)	26(57.8)		17(68.0)	16(64.0)	
Female	63(38.2)	19(42.2)		8(32.0)	9(36.0)	
**Age(y)**			0.078			0.777
≤ 60	108(65.5)	23(51.1)		14(56.0)	13(52.0)	
> 60	57(34.5)	22(48.9)		11(44.0)	12(48.0)	
**ECOG performance status**			0.288			0.733
0	133(80.6)	33(73.3)		19(76.0)	20(80.0)	
1	32(19.4)	12(26.7)		6(24.0)	5(20.0)	
**Baseline serum CEA level (ng/mL)**			0.629			1.000
≤ 5	91(55.1)	25(55.6)		16(64.0)	16(64.0)	
> 5	61(37.0)	14(31.1)		9(36.0)	9(36.0)	
NA	13(7.9)	6(13.3)		0(0.0)	0(0.0)	
**c T Stage**			0.044			0.271
2	9(5.5)	3(6.7)		0(0.0)	2(8.0)	
3	100(60.6)	18(40.0)		13(52.0)	9(36.0)	
4	56(33.9)	24(53.3)		12(48.0)	14(56.0)	
**c N Stage**			0.348			1.000
0	6(3.6)	4(8.9)		0(0.0)	1(4.0)	
1	15(9.1)	3(6.7)		2(8.0)	2(8.0)	
2	144(87.3)	38(84.4)		23(92.0)	22(88.0)	
**Tumor histologic grade**			0.316			0.725
Well/Moderate differentiated adenocarcinoma	133(80.6)	37(82.2)		20(80.0)	19(76.0)	
Poorly differentiated / Signet ring cell cancer or mucinous /adenocarcinoma	24(14.6)	8(17.8)		4(16.0)	6(24.0)	
Unknown differentiated adenocarcinoma	8(4.8)	0(0.0)		1(4.0)	0(0.0)	
**Induction chemotherapy**			0.403			0.602
No	153(92.7)	40(88.9)		24(96.0)	22(88.0)	
Yes	12(7.3)	5(11.1)		1(4.0)	3(12.0)	
**Concurrent chemotherapy**			0.175			1.000
No	1(0.6)	1(2.2)		0(0.0)	0(0.0)	
Capecitabine	139(84.2)	34(75.6)		20(80.0)	20(80.0)	
CAPEOX	25(15.2)	10(22.2)		5(20.0)	5(20.0)	
**Anterior pelvic organ invasion**			0.086			1.000
No	153(92.7)	38(84.4)		21(84.0)	21(84.0)	
Yes	12(7.3)	7(15.6)		4(16.0)	4(16.0)	
**Consolidation chemotherapy**			0.526			0.382
No	113(68.5)	28(62.2)		19(76.0)	15(60.0)	
Capecitabine	38(23.0)	14(31.1)		4(16.0)	9(36.0)	
CAPEOX	14(8.5)	3(6.7)		2(8.0)	1(4.0)	
**Time interval between neoadjuvant chemoradiotherapy and surgery, median (IQR) (weeks)**	9.7(8.1–12.0)	10.6(8.8–14.6)	0.072	8.6(7.0–11.6)	10.6(8.9–12.7)	0.062
**Preoperative serum CEA level (ng/mL)**			0.027			0.700
≤ 5	148(89.7)	37(82.2)		20(80.0)	22(88.0)	
> 5	11(6.7)	8(17.8)		5(20.0)	3(12.0)	
NA	6(3.6)	0(0)		0(0.0)	0(0.0)	
**Type of surgery**			0.016			-
Abdominoperineal resection	137(83.0)	45(100.0)		25(100.0)	25(100.0)	
Low/ultralow anterior resection	22(13.4)	0(0.0)		0(0.0)	0(0.0)	
Hartmann procedure	1(0.6)	0(0.0)		0(0.0)	0(0.0)	
Transanal total mesorectal excision	4(2.4)	0(0.0)		0(0.0)	0(0.0)	
Intersphincteric resection	1(0.6)	0(0)		0(0.0)	0(0.0)	
**Surgical technique**			0.278			0.777
Open surgery	92(55.8)	21(46.7)		13(52.0)	14(56.0)	
Laparoscopic surgery	73(44.2)	24(53.3)		12(48.0)	11(44.0)	
**Operation duration, median (IQR) (min)**	185(124–236)	208(163–273)	0.118	180(126.5–222.5)	194(105–250)	0.839
**Intraoperative blood loss, median (IQR) (ml)**	100(50–200)	100(50–100)	0.257	100(100–150)	100(50–100)	0.403
**Number of lymph nodes examined in the surgery, median (IQR)**	9(5–12)	9(4–12)	0.655	9(6.5–11)	7(4–11.5)	0.307
**Lymphovascular invasion**^a^			1.000			-
Negative	161(97.6)	43(95.6)		25(100.0)	25(100.0)	
Positive	3(1.8)	1(2.2)		0(0.0)	0(0.0)	
NA	1(0.6)	1(2.2)		0(0.0)	0(0.0)	
**Perineural invasion**^a^			0.136			1.000
Negative	153(92.7)	38(84.5)		24(96.0)	23(92.0)	
Positive	11(6.7)	6(13.3)		1(4.0)	2(8.0)	
NA	1(0.6)	1(2.2)		0(0.0)	0(0.0)	
**Resection margin**			0.220			1.000
R0	162(98.2)	42(93.3)		24(96.0)	25(100.0)	
R1	3(1.8)	3(6.7)		1(4.0)	0(0.0)	
**ypT stage**			0.807			0.229
0	40(24.2)	9(20.0)		4(16.0)	9(36.0)	
1–2	76(46.1)	21(46.7)		16(64.0)	11(44.0)	
3–4	49(29.7)	15(33.3)		5(20.0)	5(20.0)	
**ypN stage**			0.928			1.000
0	133(80.6)	36(80.0)		22(88.0)	21(84.0)	
1–2	32(19.4)	9(20.0)		3(12.0)	4(16.0)	
**Postoperative complications**			0.008			1.000
No	125(75.8)	25(55.6)		15(60.0)	16(64.0)	
Clavien- Dindo Grade I-II	32(19.4)	19(42.2)		8(32.0)	8(32.0)	
Clavien- Dindo Grade III-V	8(4.8)	1(2.2)		2(8.0)	1(4.0)	
**Adjuvant chemotherapy**			0.828			0.057
No	57(34.5)	16(35.5)		12(48.0)	11(44.0)	
Capecitabine	27(16.4)	9(20.0)		1(4.0)	7(28.0)	
CAPEOX or mFOLFOX6	68(41.2)	17(37.8)		12(48.0)	7(28.0)	
NA	13(7.9)	3(6.7)		0(0.0)	0(0.0)	

*Abbreviations*: *c* clinical, *CEA* carcinoembryonic antigen, *CRM* circumferential resection margin, *DLI* dentate line invasion, *ECOG* Eastern Cooperative Oncology Group, *IQR* interquartile range, *NA* not available, *p* pathological, *RT* radiotherapy, *SD* standardized difference, *yp* yield pathological

^a^Evaluated by postoperative pathology examination

### PSM analysis

Fifty matched patients (25 in each group) were included in the matching cohort. Table 1 also shows the clinicopathological characteristics, treatment modalities and details of the operation of the patients after appropriate PSM. No differences were found between the two groups.

### Recurrence and survival analysis

**Fig. 1:**
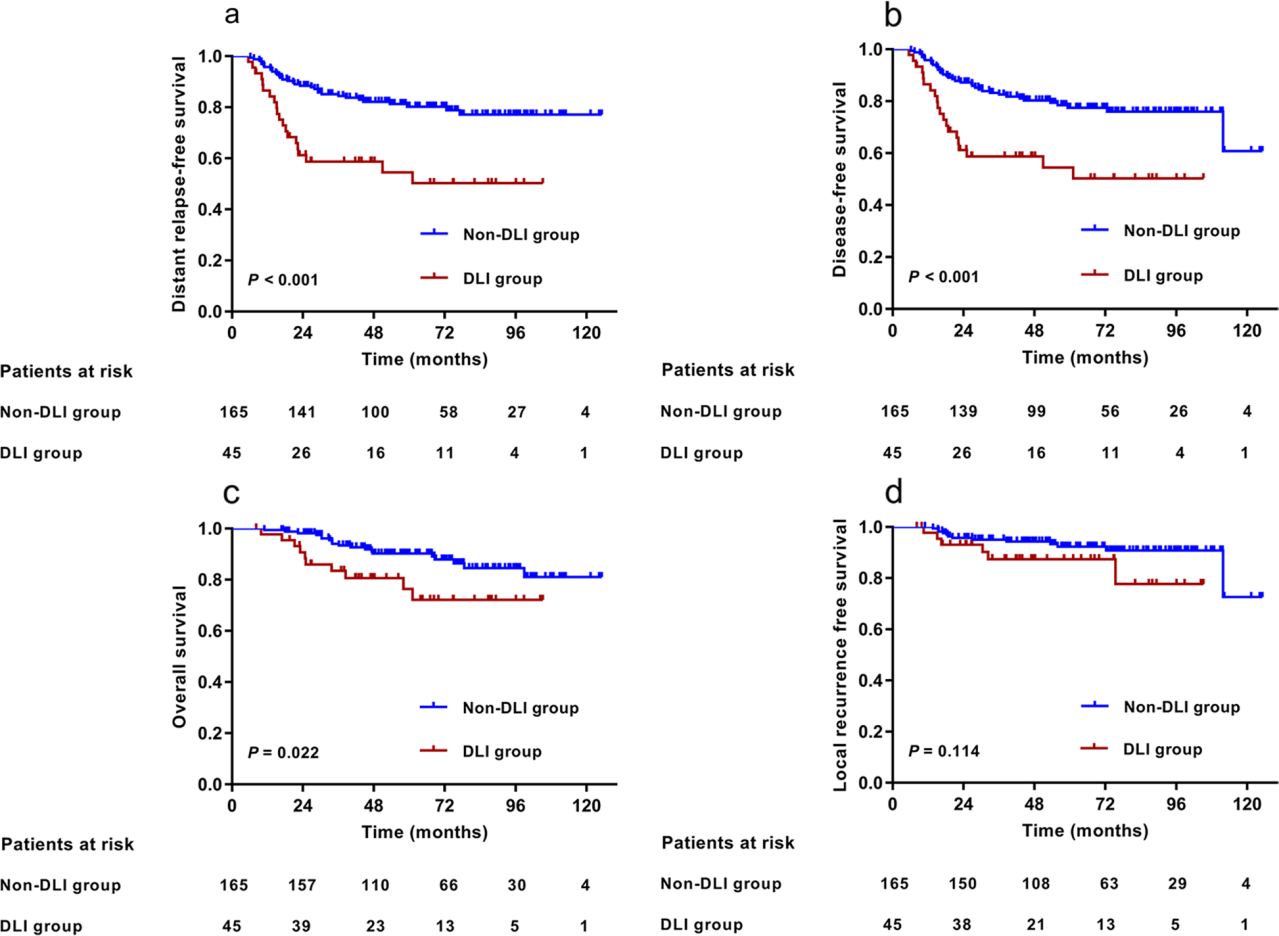
Survival analysis between DLI group (*n* = 45) and non-DLI group (*n* = 165) before propensity-score matching. **a**,**b**,**c**,**d** Kaplan‒Meier estimation of 5-year distant relapse-free survival (DRFS), disease-free survival (DFS), overall survival (OS), and local recurrence free survival (LRFS) between DLI group (*n* = 45) and non-DLI group (*n* = 165) before propensity-score matching. *Abbreviation:* DLI = dentate line invasion

Patients were followed for a median of 68.1 months (range: 8.2–125.1 months). Before matching, the 5-year DRFS for all patients was 74.9% (95% CI, 68.6%‒81.2%), 54.5% (95% CI, 38.8%–70.2%) for the DLI group, and 80.2% (95% CI, 73.7%–86.7%) for the non-DLI group (*P* < 0.001; Fig. 1a). The 5-year DFS was 72.7% (95% CI, 66.2%‒79.2%) for all patients, 54.5% (95% CI, 38.8%–70.2%) for the DLI group, and 77.4% (95% CI, 70.5%–84.3%) for the non-DLI group (*P* < 0.001; Fig. 1b). The OS rate of the whole cohort at 5 years was 87.4% (95% CI, 82.5%‒92.3%), 76.4% (95% CI, 62.3%‒90.5%) for the DLI group, and 90.2%(95% CI, 85.3%‒95.1%) for the non-DLI group (*P* = 0.022; Fig. 1c). The 5-year LRFS was 91.2% (95%CI, 87.1%‒95.3%) for the entire cohort, and there was no significant difference in LRFS between the two groups (*P* = 0.114; Fig. 1d).

**Fig. 2:**
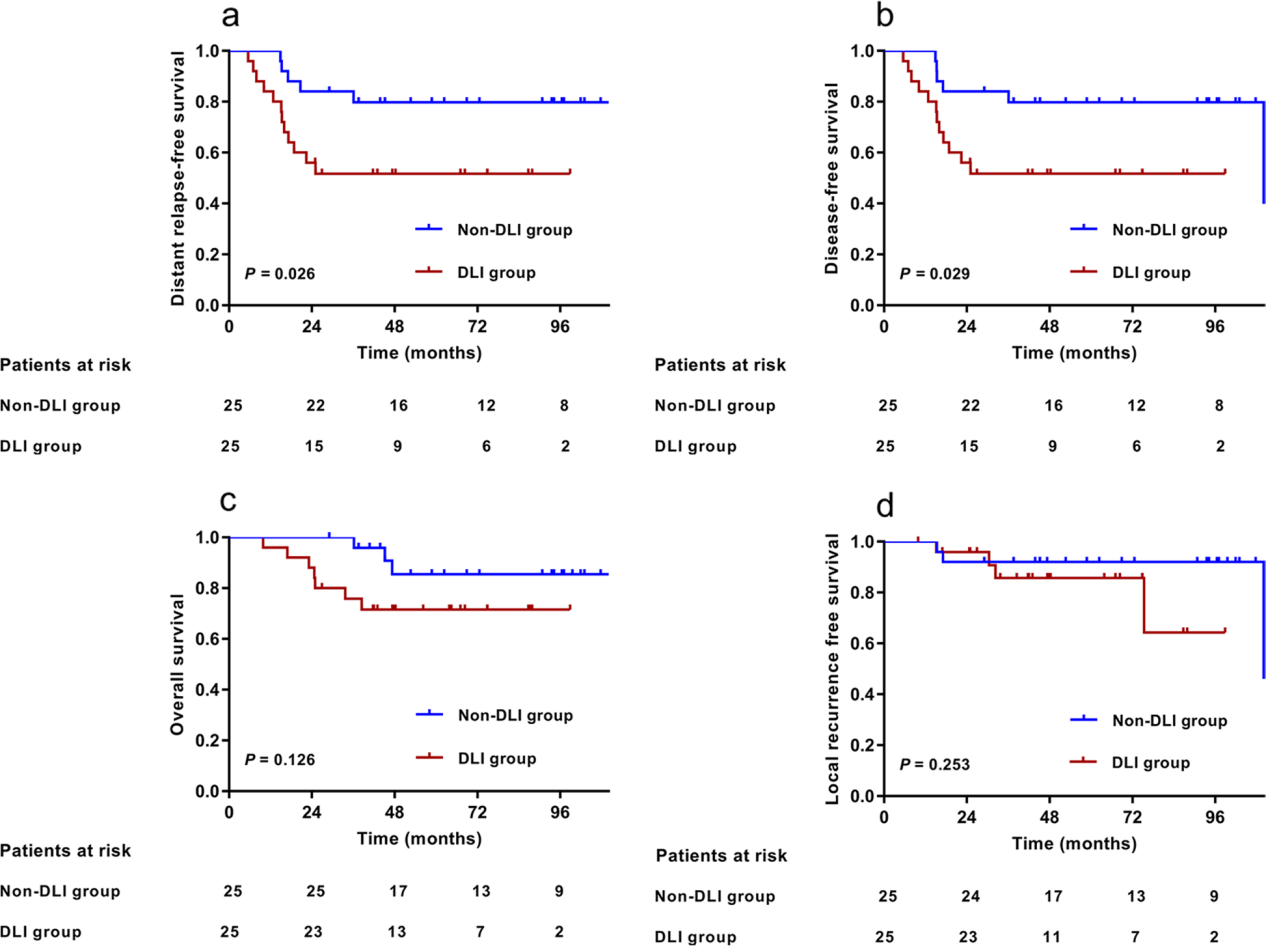
Survival analysis between DLI group (*n* = 25) and non-DLI group (*n* = 25) after propensity-score matching. **a**,**b**,**c**,**d** Kaplan‒Meier estimation of 5-year distant relapse-free survival (DRFS), disease-free survival (DFS), overall survival (OS), and local recurrence free survival (LRFS) between DLI group (*n* = 25) and non-DLI group (*n* = 25) after propensity-score matching. *Abbreviation:* DLI = dentate line invasion

After PSM, the median duration of follow-up was 68.3 months (range: 26.9–121.4 months). The 5-year DRFS, DFS, OS, and LRFS were 51.7% (95%CI, 31.9%–71.5%) vs. 79.8%(95%CI, 63.9%–95.7%)(*P* = 0.026; Fig. 2a), 51.7% (95%CI, 31.9%–71.5%) vs. 79.8%(95%CI, 63.9%–95.7%)(*P* = 0.029; Fig. 2b), 71.6% (95%CI, 53.8%–89.4%) vs. 85.4%(95%CI, 70.1%–100.0%)(*P* = 0.126; Fig. 2c), and 85.7% (95%CI, 70.6%–100.0%) vs. 92.0% (95% CI, 81.4%‒100%)(*P* = 0.253; Fig. 2d) for the DLI group vs. non-DLI group, respectively.

**Fig. 3:**
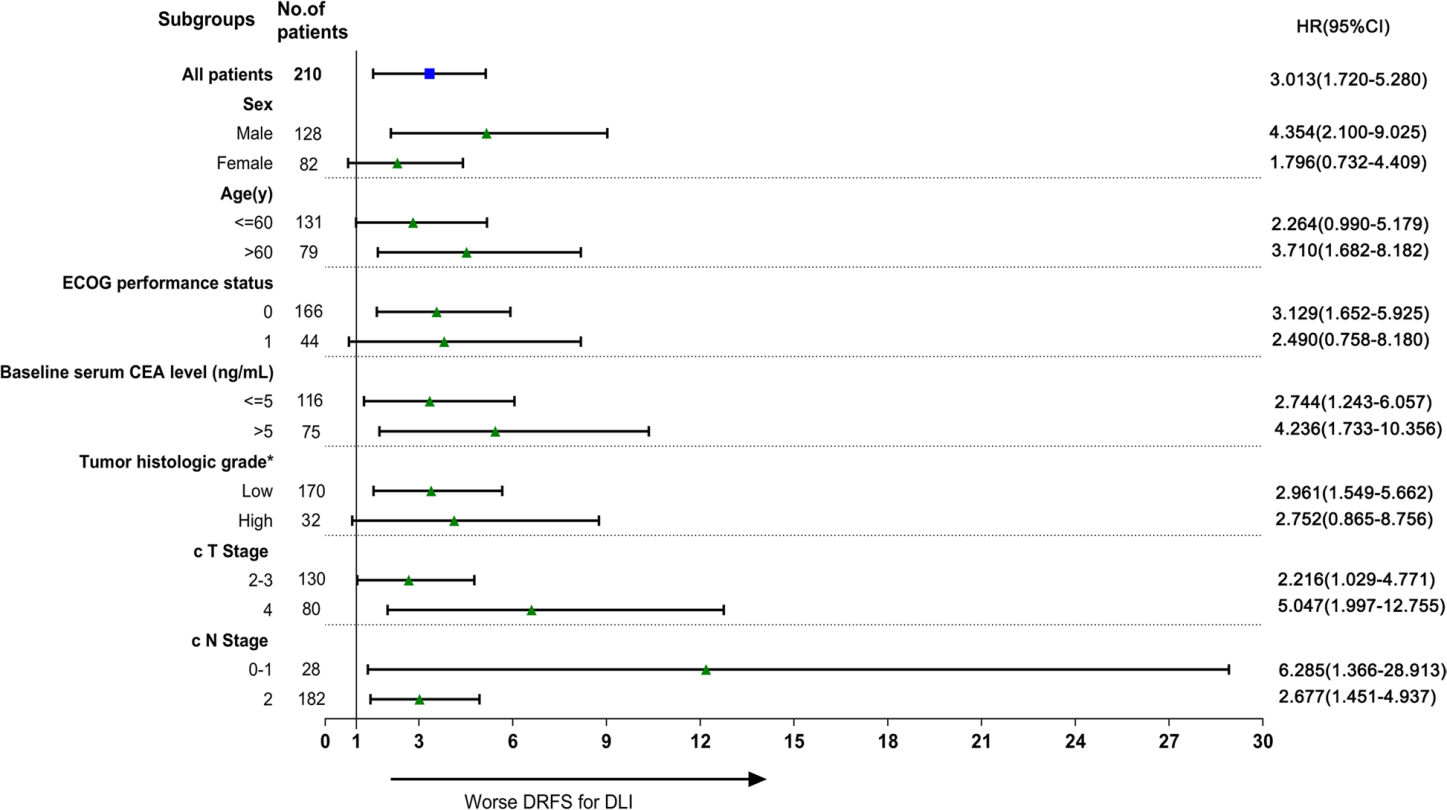
Forest plot of HRs for DLI versus non-DLI of LALRC with anal sphincter involvement in the subgroup analysis of distant relapse-free survival. *Abbreviations:* HRs = hazard ratios, DLI = dentate line invasion, DRFS = distant relapse-free survival, LALRC = locally advanced lower rectal cancer, CEA = carcinoembryonic antigen, c = clinical, ECOG = Eastern Cooperative Oncology Group. *Evaluated by pretreatment diagnostic biopsy. Low indicates well or moderately differentiated; high indicates poorly differentiated, mucinous, or signet ring cell carcinoma

For the whole cohort, DLI was an independent prognostic factor for worse DRFS (HR 2.567, 95%CI 1.438–4.582, *P* = 0.001) (Supplementary Table 1) and DFS (HR 2.344, 95%CI 1.325–4.146, *P* = 0.003) (Supplementary Table 2) but not for OS (HR 1.707, 95%CI 0.730–3.995, *P* = 0.217) (Supplementary Table 3). Subgroup analyses for variables including sex, age, ECOG performance status, baseline serum CEA level, tumor histologic grade, and cT and cN stage were also performed to further investigate the pre-treatment significance of DLI for DRFS. Figure 3 shows the obtained forest plot. However, the presence of DLI was associated with a lower rate of DRFS in most subgroups.

For the matched patients, the independent poor prognostic factors for DRFS were tumors in which the lower edge had DLI (HR 3.843, 95% CI 1.236–11.949, *P* = 0.020), and R1 resection (HR 22.029, 95% CI 2.158–224.903, *P* = 0.009) (Supplementary Table 4). Similarly, DLI was also an independent prognostic factor for DFS (HR 3.765, 95% CI 1.211–11.704, *P* = 0.022)(Supplementary Table 5).

### The failure patterns

Before matching, the 5-year distant metastasis rate was 24.8% (52/210) in the whole cohort, and the most common metastatic sites were the lungs (15.2%) followed by the liver (6.2%) and bone (5.7%). Patients with DLI showed significantly higher rates of distant metastasis (44.4% vs. 19.4%, *P* < 0.001) than those without DLI, particularly in the lungs, bone, and inguinal and retroperitoneal lymph nodes. Distant relapse was detected in six patients (30%) in the first year, 11 (55%) in the second year, 2(10%) in the third to fifth years, and only one patient (5%) diagnosed with recurrence after 5 years in the DLI group. The 5-year locoregional recurrence rate was 9.0% (19/210) for the entire cohort. The most common locoregional recurrence sites were the anterior and lateral pelvic subsites, and the locoregional recurrence rate was similar in the remaining subsites. Comparing the DLI and non-DLI groups, the 5-year locoregional recurrence rates were 13.3% (6/45) and 7.9% (13/165)(*P* = 0.729), respectively. Detailed failure patterns before matching are listed in Table 2.

**Table 2:** The failure patterns before matching

Failure site	Total group (*n* = 210) n (%)	Non-DLI group (*n* = 165) n (%)	DLI group (*n* = 45) n (%)	*P*
**Distant metastasis**	52(24.8)	32(19.4)	20(44.4)	** < 0.001**
External iliac lymph node	7(3.3)	5(3.0)	2(4.4)	0.996
Inguinal lymph node	10(4.8)	5(3.0)	5(11.1)	**0.024**
Retroperitoneal lymph node	10(4.8)	5(3.0)	5(11.1)	**0.024**
Common iliac lymph nodes	2(1.0)	2(1.2)	0(0.0)	0.902
Lungs	32(15.2)	19(11.5)	13(28.9)	**0.004**
Liver	13(6.2)	8(4.8)	5(11.1)	0.122
Bone	12(5.7)	6(3.6)	6(13.3)	**0.013**
Brain	4(1.9)	2(1.2)	2(4.4)	0.426
Peritoneum	2(1.0)	1(0.6)	1(2.2)	0.900
Adrenal gland	2(1.0)	1(0.6)	1(2.2)	0.900
Ovary	1(0.5)	0(0.0)	1(2.2)	0.214
Pleura	1(0.5)	1(0.6)	0(0.0)	1.000
Left supraclavicular lymph node	3(1.4)	2(1.2)	1(2.2)	0.840
Mediastinal lymph nodes	4(1.9)	2(1.2)	2(4.4)	0.426
Pericardium	1(0.5)	0(0.0)	1(2.2)	0.214
Pancreas	1(0.5)	1(0.6)	0(0.0)	1.000
Other sites	3(1.4)	3(1.8)	0(0.0)	0.840
Unknow	3(1.4)	2(1.2)	1(2.2)	-
**Locoregional recurrence**	19(9.0)	13(7.9)	6(13.3)	0.729
Anterior pelvic subsite	7(3.3)	4(2.4)	3(6.7)	0.349
Posterior pelvic subsite	3(1.4)	3(1.8)	0(0.0)	0.840
Lateral pelvic subsite	6(2.9)	3(1.8)	3(6.7)	0.220
Inferior pelvic subsite	3(1.4)	2(1.2)	1(2.2)	0.840
Surgical anastomotic subsite	2(1.0)	2(1.2)	0(0.0)	0.902

One patients eventually died without clear locoregional recurrence or distant metastasis

*Abbreviation*: *DLI* Dentate line invasion

After PSM, patients with DLI had a higher rate of distant metastasis (48.0% vs. 20.0%, *P* = 0.037) than those without DLI. Distant metastasis sites such as the lungs and retroperitoneal lymph nodes tended to occur in patients with DLI. Moreover, the 5-year locoregional recurrence rates in the DLI and non-DLI groups were 16.0% (4/25) and 12.0% (3/25)(*P* = 1.000), respectively (Table 3).

**Table 3:** The failure patterns after matching

Failure site	Total group (*n* = 50) n (%)	Non-DLI group (*n* = 25)n (%)	DLI group (*n* = 25) n (%)	*P*
**Distant metastasis**	17(34.0)	5(20.0)	12(48.0)	**0.037**
External iliac lymph node	3(6.0)	1(4.0)	2(8.0)	1.000
Inguinal lymph node	6(12.0)	2(8.0)	4(16.0)	0.663
Retroperitoneal lymph node	6 (12.0)	1(4.0)	5(20.0)	0.192
Common iliac lymph nodes	1(2.0)	1(4.0)	0(0.0)	1.000
Lungs	12(24.0)	3(12.0)	9(36.0)	0.098
Liver	4(8.0)	1(4.0)	3(12.0)	0.602
Bone	2(4.0)	0(0.0)	2(8.0)	0.490
Brain	2(4.0)	0(0.0)	2(8.0)	0.490
Peritoneum	1(2.0)	0(0.0)	1(4.0)	1.000
Adrenal gland	1(2.0)	0(0.0)	1(4.0)	1.000
Ovary	1(2.0)	0(0.0)	1(4.0)	1.000
Pleura	0(0.0)	0(0.0)	0(0.0)	-
Left supraclavicular lymph node	1(2.0)	1(4.0)	0(0.0)	1.000
Mediastinal lymph nodes	2(4.0)	1(4.0)	1(4.0)	1.000
Pericardium	1(2.0)	0(0.0)	1(4.0)	1.000
**Locoregional recurrence**	7(14.0)	3(12.0)	4(16.0)	1.000
Anterior pelvic subsite	3(6.0)	1(4.0)	2(8.0)	1.000
Posterior pelvic subsite	0(0.0)	0(0.0)	0(0.0)	-
Lateral pelvic subsite	2(4.0)	0(0.0)	2(8.0)	0.490
Inferior pelvic subsite	3(6.0)	2(8.0)	1(4.0)	1.000
Surgical anastomotic subsite	-	-	-	-

One patients eventually died without locoregional recurrence or distant metastasis

*Abbreviation*: *DLI* Dentate line invasion

Among the 32 patients with lung metastases before matching, five (15.6%) had solitary lung metastases, 25 (78.1%) presented with multiple lung metastases, and two (6.3%) did not comment on the results. There were no statistically significant differences in the incidence rates of solitary and multiple lung metastasis between the two groups(*P* = 0.138). The median time interval from the first day of NCRT to the development of lung metastases was 19.0 months, and it was shorter for patients with DLI than for those without (15.1 vs. 23.1 months, *P* = 0.042). Lung relapse was detected in six patients (18.8%) in the first year, 16 (50%) in the second year, 8 (25%) in the third to fifth years, and in only two patients (6.3%) diagnosed with recurrence after 5 years. Among the matched patients, there were no statistical differences in solitary and multiple lung metastasis rates( *P* = 1.000) nor median time interval from the first day of NCRT to the development of lung metastases (15.3 vs. 15.1 months, *P* = 0.644) between the two groups.

## Discussion

While an important surgical landmark of the dentate line has been established for LALRC, the prognostic significance of DLI has not been well defined. Our real-world cohort study comprehensively explored the exact role of DLI in survival and failure patterns in LALRC patients with anal sphincter involvement both before and after PSM. This study demonstrated that DLI in the lower edge of tumors significantly affected DRFS and distant metastasis in LALRC patients with anal sphincter involvement after NCRT followed by TME. Distant failure remains a greater challenge for patients with DLI than for those without invasion. Furthermore, we are convinced that the present results are useful for the evaluation of clinical treatment decisions including organ preservation strategies in LALRC [27].

Many factors influence the survival of LALRC patients, but DLI has seldom been explored. To our knowledge, this was the largest single-center real-world PSM study and was adjusted for selection bias in an observational study of causal effects. Our results showed that DLI is an independent prognostic factor that affects DRFS and DFS but not for LRFS and OS for these patients. However, a Japanese study showed that patients with DLI showed a significantly higher local recurrence rate and worse OS, but this study did not use the Cox proportional hazard multivariate model to analyze the effects on survival, especially DRFS or DFS [16]. Besides, Shiratori et al. have alternatively drawn the conclusion that DLI was not associated with survival [28]. There are various reasons for these inconsistent results including different study populations, limited sample sizes, confounding biases between groups, and the heterogeneity of perioperative treatment modalities in the early era. In our study, we included a larger sample size of ultra LALRC patients who underwent neoadjuvant long-course chemoradiation and standard TME, which means that the risk of bias associated with unknown variables was minimized by lower clinical heterogeneity. More importantly, we also performed a reasonably controlled PSM study based on the Cox proportional hazard multivariate model because more standard prognostic factors were considered in the analysis.

In this study, our results showed that patients with DLI had a much higher incidence of distant metastasis than those without invasion both before and after matching. The significant increase in distant metastases in the DLI group may be multifactorial rather than related to locoregional recurrence as there was no significant increase in locoregional failures. Distant metastasis in lungs, liver, bone, inguinal and retroperitoneal lymph nodes occurred more in the DLI group, and some sites tended to occur even after matching. These increased distant metastasis sites may be explained by the following anatomical factors. First, the lymphatics below the dentate line mainly drain into the ILNs, and some recent clinical studies have confirmed that patients with DLI have a higher rate ILN failure [17, 28]. In addition, the superior rectal vein drains the rectum and anal canal above the dentate line through the inferior mesenteric vein to the portal venous system, and the external hemorrhoid plexus is below the dentate line, which drains by way of the inferior rectal vein to the internal pudendal vein and then into the internal iliac vein and systemic circulation [29]. Therefore, rectal tumors below the dentate line may easily metastasize initially to the lungs and other distant sites because the inferior rectal vein finally drains into the inferior vena cava, bypassing the portal venous system and facilitating access to the systemic venous circulation [8]. The phenomenon of different metastasis patterns based on the location of colorectal cancer has also been confirmed in other clinical studies [8, 30, 31]. Because the intensification of neoadjuvant chemotherapy has the potential to reduce systemic relapses, it might provide new focus to deal with treatment of tumors with DLI, as systemic venous circulation plays a more important role in the metastatic process [10, 32]. Moreover, the failure patterns dominated by distant metastasis also support the need for intensified surveillance within two years to allow for better prognosis and longer survival for LALRC patients with DLI.

Although ILNs and external iliac lymph nodes (ELNs) are recommended for irradiation during NCRT in most of the current guidelines, it is unclear whether these regions need to be included in the CTV because metastases of ILNs and ELNs seem to be relatively rare [16, 28, 33–42]. Meanwhile, irradiation of ILNs and ELNs might result in significant and frequent complications because of the larger treatment volume. This study showed that the 5-year failure rate was 4.8% for ILNs and 3.3% for ELNs, which were as low as our previous 3-year failure rates [17]. In addition, the anatomical mechanism and failure patterns also indicated that ILNs and ELNs might be systemic metastatic stations [17, 43]. Hence, irradiation of ILNs and ELNs may not potentially improve the prognosis of all patients to some extent. Before matching, the 5-year ILN failure rate was significantly higher in the DLI group; however, the difference was not statistically significant after matching. Considering the limited number of patients after matching, it remains uncertain whether the presence of tumors with DLI is an indication for ILN irradiation.

Our study had some limitations. First, this was a retrospective single-center cohort study, which could have introduced other potentially unmeasured confounding biases, such as missing data. Second, the sample size of the study cohort was small after 1:1 PSM. Further prospective studies with a larger number of participants are required to confirm these findings. Third, because the structure of the anal canal is very detailed, MRI examination is sometimes subtle in assessing anal sphincter involvement.

## Conclusions

In conclusion, DLI in the lower edge of tumors may be a prognostic indicator of worse DRFS and distant metastasis for LALRC patients with anal sphincter involvement. DLI may be an important variable that can help guide clinicians in the management of patients. A multicenter prospective study is needed to confirm our findings.

## Supplementary Information

**Additional file 1: Supplementary Table 1.** Univariate and multivariate Cox proportional hazards model for DRFS before matching. **Supplementary Table 2.** Univariate and multivariate Cox proportional hazards model for DFS before matching. **Supplementary Table 3.** Univariate and multivariate Cox proportional hazards model for OS before matching. **Supplementary Table 4.** Univariate and multivariate Cox proportional hazards model for DRFS after matching. **Supplementary Table 5.** Univariate and multivariate Cox proportional hazards model for DFS after matching.

## Data Availability

The statistical datasets and materials used and/or analyzed in the current study are available from the corresponding author (wangweihu88@163.com) on reasonable request.

## Abbreviations

CEA: Carcinoembryonic antigen
cT and cN: Clinical T and N stages
CT: Computed Tomography
CTV: Clinical target volume
DFS: Disease-free survival
DLI: Dentate line invasion
DRFS: Distant relapse-free survival
ECOG: Eastern Cooperative Oncology Group
ELN: External iliac lymph node
ILN: Inguinal lymph node
LALRC: Locally advanced lower rectal cancer
LRFS: Local recurrence-free survival
MRI: Magnetic resonance imaging
NCRT: Neoadjuvant chemoradiotherapy
PSM: Propensity-score matching
TME: Total mesorectal excision
