# Supplementary material for: Dentate line invasion as a predictive factor of poor distant relapse-free survival in locally advanced lower rectal cancer with anal sphincter involvement
Source: BMC Cancer. 2022 Nov 19;22:1196. doi: 10.1186/s12885-022-10299-8 (PMC9675199; doi:10.1186/s12885-022-10299-8)
Supplement: Supplementary file 1 — Additional file 1: Supplementary Table 1. Univariate and multivariate Cox proportional hazards model for DRFS before matching. Supplementary Table 2. Univariate and multivariate Cox proportional hazards model for DFS before matching. Supplementary Table 3. Univariate and multivariate Cox proportional hazards model for OS before matching. Supplementary Table 4. Univariate and multivariate Cox proportional hazards model for DRFS after matching. Supplementary Table 5. Univariate and multivariate Cox proportional hazards model for DFS after matching. [file 12885_2022_10299_MOESM1_ESM.docx]

| **Supplementary Table 1:** Univariate and multivariate Cox proportional hazards model for DRFS before matching. | | | | |
| --- | --- | --- | --- | --- |
| Variable | Univariate | | Multivariate | |
|  | HR (95% CI) | *P* | HR (95% CI) | *P* |
| Sex (male vs. female) | 0.902(0.520-1.564) | 0.714 |  |  |
| Age (y) (>60 vs. ≤60) | 1.721(0.997-2.970) | 0.051 |  |  |
| ECOG (1 vs. 0) | 1.103(0.566-2.147) | 0.774 |  |  |
| Baseline serum CEA level (ng/mL) (>5 vs. ≤5) | 1.465(0.836-2.568) | 0.182 |  |  |
| Tumor histological grade* |  |  |  |  |
| Low | 1 |  |  |  |
| High | 1.731(0.906-3.309) | 0.097 |  |  |
| Clinical anal sphincter status at diagnosis |  |  |  |  |
| Tumor invades internal anal sphincter | 1 |  |  |  |
| Tumor invades both internal and external sphincter | 1.174(0.615-2.241) | 0.626 |  |  |
| Lower edge of tumors with the dentate line invasion(yes vs. no) | 3.013(1.720-5.280) | <0.001 | 2.567(1.438-4.582) | 0.001 |
| c T Stage |  |  |  |  |
| 2 | 1 |  |  |  |
| 3 | 3.291(0.449-24.096) | 0.241 |  |  |
| 4 | 2.892(0.387-21.607) | 0.301 |  |  |
| c N Stage |  |  |  |  |
| 0 | 1 |  |  |  |
| 1 | 0.700(0.157-3.129) | 0.641 |  |  |
| 2 | 0.767(0.238-2.472) | 0.657 |  |  |
| Induction chemotherapy (yes vs. no) | 1.210(0.433-3.377) | 0.716 |  |  |
| Concurrent chemotherapy |  |  |  |  |
| No | 1 |  | 1 |  |
| Capecitabine | 0.107(0.025-0.450) | 0.002 | 0.233(0.052-1.049) | 0.058 |
| CAPEOX | 0.120(0.025-0.571) | 0.008 | 0.215(0.042-1.107) | 0.066 |
| Consolidation chemotherapy |  |  |  |  |
| No | 1 |  |  |  |
| Capecitabine | 0.766(0.380-1.544) | 0.456 |  |  |
| CAPEOX | 1.052(0.373-2.969) | 0.924 |  |  |
| Preoperative serum CEA level (ng/mL) (>5 vs. ≤5) | 3.244(1.622-6.488) | 0.001 | 2.237(1.089-4.598) | 0.028 |
| Resection margin (R1 vs. R0) | 7.179(2.832-18.199) | <0.001 | 1.299(0.331-5.096) | 0.707 |
| yp T Stage(3-4 vs. 0-2) | 2.441(1.415-4.210) | 0.001 | 1.973(1.109-3.509) | 0.021 |
| yp N Stage(N+ vs. N0) | 3.222(1.841-5.639) | <0.001 | 2.611(1.436-4.750) | 0.002 |
| ypCR(yes vs. no) | 0.281(0.101-0.780) | 0.015 | 0.584(0.195-1.749) | 0.336 |
| Lymphovascular invasion(positive vs. negative) | 5.182(1.604-16.745) | 0.006 | 1.675(0.428-6.561) | 0.459 |
| Perineural invasion(positive vs. negative) | 3.018(1.461-6.232) | 0.003 | 1.734(0.756-3.976) | 0.194 |
| Adjuvant chemotherapy |  |  |  |  |
| No | 1 |  |  |  |
| Capecitabine | 0.960(0.428-2.157) | 0.922 |  |  |
| CAPEOX or mFOLFOX6 | 1.040(0.548-1.973) | 0.905 |  |  |
| Type of surgery |  |  |  |  |
| Abdominoperineal resection | 1 |  |  |  |
| Low/ultralow anterior resection | 1.078(0.458-2.535) | 0.864 |  |  |
| Hartmann procedure | 6.397(0.870-47.035) | 0.068 |  |  |
| Transanal total mesorectal excision | 2.803(0.675-11.639) | 0.156 |  |  |
| Intersphincteric resection | - | 0.978 |  |  |
| Surgical technique **(**Laparoscopic vs. Open surgery) | 1.004(0.579-1.739) | 0.989 |  |  |
| Operation duration(min) | 0.999(0.996-1.003) | 0.690 |  |  |
| Intraoperative blood loss (ml) (>100 vs.≤100) | 1.083(0.601-1.951) | 0.792 |  |  |
| Number of lymph nodes examined in the surgery | 0.960(0.909-1.014) | 0.147 |  |  |
| Postoperative complications |  |  |  |  |
| No |  |  |  |  |
| Clavien- Dindo Grade I-II | 1.521(0.839-2.758) | 0.167 |  |  |
| Clavien- Dindo Grade III-V | 0.997(0.239-4.150) | 0.996 |  |  |

Abbreviations: CEA=carcinoembryonic antigen; c=clinical; DRFS= distant relapse-free survival; ECOG=Eastern Cooperative Oncology Group; pCR=pathologic complete response; RT=radiotherapy; yp=yield pathological;

*Evaluated by pretreatment diagnostic biopsy. Low indicates well or moderately differentiated; high indicates poorly differentiated, mucinous, or signet ring cell carcinoma.

| **Supplementary Table 2:** Univariate and multivariate Cox proportional hazards model for DFS before matching. | | | | |
| --- | --- | --- | --- | --- |
| Variable | Univariate | | Multivariate | |
|  | HR (95% CI) | *P* | HR (95% CI) | *P* |
| Sex (male vs. female) | 0.960(0.563-1.636) | 0.880 |  |  |
| Age (y) (>60 vs. ≤60) | 1.679(0.992-2.843) | 0.054 |  |  |
| ECOG (1 vs. 0) | 1.192(0.637-2.230) | 0.583 |  |  |
| Baseline serum CEA level (ng/mL) (>5 vs. ≤5) | 1.675(0.973-2.886) | 0.063 |  |  |
| Tumor histological grade* |  |  |  |  |
| Low | 1 |  |  |  |
| High | 1.602(0.843-3.044) | 0.151 |  |  |
| Clinical anal sphincter status at diagnosis |  |  |  |  |
| Tumor invades internal anal sphincter | 1 |  |  |  |
| Tumor invades both internal and external sphincter | 1.189(0.638-2.216) | 0.585 |  |  |
| Lower edge of tumors with the dentate line invasion(yes vs. no) | 2.737(1.577-4.750) | 0.000 | 2.344(1.325-4.146) | 0.003 |
| c T Stage |  |  |  |  |
| 2 | 1 |  |  |  |
| 3 | 3.416(0.467-24.972) | 0.226 |  |  |
| 4 | 3.189(0.429-23.720) | 0.257 |  |  |
| c N Stage |  |  |  |  |
| 0 | 1 |  |  |  |
| 1 | 0.700(0.157-3.128) | 0.641 |  |  |
| 2 | 0.832(0.259-2.672) | 0.757 |  |  |
| Induction chemotherapy (yes vs. no) | 1.438(0.570-3.631) | 0.442 |  |  |
| Concurrent chemotherapy |  |  |  |  |
| No | 1 |  | 1 |  |
| Capecitabine | 0.108(0.026-0.455) | 0.002 | 0.240(0.053-1.094) | 0.065 |
| CAPEOX | 0.149(0.032-0.688) | 0.015 | 0.292(0.058-1.480) | 0.137 |
| Consolidation chemotherapy |  |  |  |  |
| No | 1 |  |  |  |
| Capecitabine | 0.802(0.409-1.571) | 0.519 |  |  |
| CAPEOX | 1.318(0.516-3.368) | 0.564 |  |  |
| Preoperative serum CEA level (ng/mL) (>5 vs. ≤5) | 2.974(1.495-5.917) | 0.002 | 2.117(1.037-4.322) | 0.040 |
| Resection margin (R1 vs. R0) | 6.680(2.647-16.860) | <0.001 | 1.310(0.340-5.046) | 0.695 |
| yp T Stage(3-4 vs. 0-2) | 2.276(1.345-3.852) | 0.002 | 1.879(1.078-3.277) | 0.026 |
| yp N Stage(N+ vs. N0) | 2.949(1.701-5.113) | <0.001 | 2.438(1.355-4.387) | 0.003 |
| ypCR(yes vs. no) | 0.259(0.094-0.718) | 0.009 | 0.462(0.157-1.359) | 0.161 |
| Lymphovascular invasion(positive vs. negative) | 4.904(1.522-15.806) | 0.008 | 1.874(0.486-7.218) | 0.362 |
| Perineural invasion(positive vs. negative) | 2.815(1.371-5.778) | 0.005 | 1.606(0.704-3.661) | 0.260 |
| Adjuvant chemotherapy |  |  |  |  |
| No | 1 |  |  |  |
| Capecitabine | 1.083(0.496-2.368) | 0.841 |  |  |
| CAPEOX or mFOLFOX6 | 1.146(0.611-2.147) | 0.671 |  |  |
| Type of surgery |  |  |  |  |
| Abdominoperineal resection | 1 |  |  |  |
| Low/ultralow anterior resection | 1.262(0.569-2.799) | 0.567 |  |  |
| Hartmann procedure | 6.071(0.827-44.561) | 0.076 |  |  |
| Transanal total mesorectal excision | 2.665(0.643-11.046) | 0.177 |  |  |
| Intersphincteric resection | - | 0.977 |  |  |
| Surgical technique **(**Laparoscopic vs. Open surgery) | 0.970(0.568-1.655) | 0.910 |  |  |
| Operation duration(min) | 1.000(0.996-1.003) | 0.859 |  |  |
| Intraoperative blood loss (ml) (>100 vs.≤100) | 1.025(0.579-1.816) | 0.932 |  |  |
| Number of lymph nodes examined in the surgery | 0.956(0.906-1.009) | 0.102 |  |  |
| Postoperative complications |  |  |  |  |
| No | 1 |  |  |  |
| Clavien- Dindo Grade I-II | 1.485(0.831-2.656) | 0.182 |  |  |
| Clavien- Dindo Grade III-V | 1.545(0.475-5.021) | 0.470 |  |  |

Abbreviations: CEA=carcinoembryonic antigen; c=clinical; DFS=disease-free survival; ECOG=Eastern Cooperative Oncology Group; pCR=pathologic complete response; RT=radiotherapy; yp=yield pathological;

*Evaluated by pretreatment diagnostic biopsy. Low indicates well or moderately differentiated; high indicates poorly differentiated, mucinous, or signet ring cell carcinoma.

| **Supplementary Table 3:** Univariate and multivariate Cox proportional hazards model for OS before matching. | | | | |
| --- | --- | --- | --- | --- |
| Variable | Univariate | | Multivariate | |
|  | HR (95% CI) | *P* | HR (95% CI) | *P* |
| Sex (male vs. female) | 1.207(0.569-2.560) | 0.624 |  |  |
| Age (y) (>60 vs. ≤60) | 1.804(0.869-3.745) | 0.113 |  |  |
| ECOG (1 vs. 0) | 1.080(0.438-2.662) | 0.867 |  |  |
| Baseline serum CEA level (ng/mL) (>5 vs. ≤5) | 1.722(0.820-3.615) | 0.151 |  |  |
| Tumor histological grade* |  |  |  |  |
| Low | 1 |  |  |  |
| High | 1.743(0.740-4.105) | 0.203 |  |  |
| Clinical anal sphincter status at diagnosis |  |  |  |  |
| Tumor invades internal anal sphincter | 1 |  |  |  |
| Tumor invades both internal and external sphincter | 1.111(0.471-2.618) | 0.810 |  |  |
| Lower edge of tumors with the dentate line invasion(yes vs. no) | 2.386(1.106-5.148) | 0.027 | 1.707(0.730-3.995) | 0.217 |
| c T Stage |  |  |  |  |
| 2 | 1 |  |  |  |
| 3 | 0.421(0.121-1.470) | 0.175 |  |  |
| 4 | 0.517(0.145-1.836) | 0.307 |  |  |
| c N Stage |  |  |  |  |
| 0 | 1 |  |  |  |
| 1 | 0.936(0.156-5.614) | 0.942 |  |  |
| 2 | 0.564(0.133-2.391) | 0.437 |  |  |
| Induction chemotherapy (yes vs. no) | 1.552(0.360-6.685) | 0.555 |  |  |
| Concurrent chemotherapy |  |  |  |  |
| No | 1 |  | 1 |  |
| Capecitabine | 0.084(0.011-0.651) | 0.018 | 0.322(0.034-3.069) | 0.324 |
| CAPEOX | 0.099(0.011-0.907) | 0.041 | 0.333(0.029-3.865) | 0.379 |
| Consolidation chemotherapy |  |  |  |  |
| No | 1 |  |  |  |
| Capecitabine | 0.593(0.202-1.737) | 0.340 |  |  |
| CAPEOX | 0.573(0.076-4.310) | 0.589 |  |  |
| Preoperative serum CEA level (ng/mL) (>5 vs. ≤5) | 4.654(1.960-11.054) | <0.001 | 3.410(1.340-8.678) | 0.010 |
| Resection margin (R1 vs. R0) | 11.539(3.909-34.063) | <0.001 | 3.740(1.102-12.694) | 0.034 |
| yp T Stage(3-4 vs. 0-2) | 3.171(1.523-6.602) | 0.002 | 2.649(1.189-5.905) | 0.017 |
| yp N Stage(N+ vs. N0) | 2.379(1.104-5.129) | 0.027 | 1.463(0.618-3.461) | 0.387 |
| ypCR(yes vs. no) | 0.789(0.300-2.072) | 0.630 | 0.462(0.157-1.359) | 0.161 |
| Lymphovascular invasion(positive vs. negative) | 7.270(1.688-31.313) | 0.008 | 3.853(0.835-17.768) | 0.084 |
| Perineural invasion(positive vs. negative) | 3.445(1.290-9.204) | 0.014 | 0.757(0.165-3.480) | 0.720 |
| Adjuvant chemotherapy |  |  |  |  |
| No | 1 |  |  |  |
| Capecitabine | 0.469(0.128-1.712) | 0.252 |  |  |
| CAPEOX or mFOLFOX6 | 0.705(0.290-1.711) | 0.439 |  |  |
| Type of surgery |  |  |  |  |
| Abdominoperineal resection | 1 |  |  |  |
| Low/ultralow anterior resection | 0.238(0.032-1.758) | 0.159 |  |  |
| Hartmann procedure | - | 0.986 |  |  |
| Transanal total mesorectal excision | 2.739(0.366-20.480) | 0.326 |  |  |
| Intersphincteric resection | - | 0.988 |  |  |
| Surgical technique (Laparoscopic vs. Open surgery) | 1.150(0.541-2.444) | 0.716 |  |  |
| Operation duration(min) | 1.001(0.996-1.006) | 0.794 |  |  |
| Intraoperative blood loss (ml) (>100 vs.≤100) | 1.005(0.457-2.210) | 0.989 |  |  |
| Number of lymph nodes examined in the surgery | 0.927(0.856-1.005) | 0.066 |  |  |
| Postoperative complications |  |  |  |  |
| No | 1 |  |  |  |
| Clavien- Dindo Grade I-II | 1.343(0.587-3.073) | 0.485 |  |  |
| Clavien- Dindo Grade III-V | 1.846(0.429-7.941) | 0.410 |  |  |

Abbreviations: CEA=carcinoembryonic antigen; c=clinical; ECOG=Eastern Cooperative Oncology Group; OS= overall survival;pCR=pathologic complete response; RT=radiotherapy; yp=yield pathological;

*Evaluated by pretreatment diagnostic biopsy. Low indicates well or moderately differentiated; high indicates poorly differentiated, mucinous, or signet ring cell carcinoma.

| **Supplementary Table 4:** Univariate and multivariate Cox proportional hazards model for DRFS after matching. | | | | |
| --- | --- | --- | --- | --- |
| Variable | Univariate | | Multivariate | |
|  | HR (95% CI) | *P* | HR (95% CI) | *P* |
| Sex (male vs. female) | 0.985(0.364-2.664) | 0.976 |  |  |
| Age (y) (>60 vs. ≤60) | 1.422(0.548-3.687) | 0.469 |  |  |
| ECOG (1 vs. 0) | 0.714(0.205-2.485) | 0.596 |  |  |
| Baseline serum CEA level (ng/mL) (>5 vs. ≤5) | 1.797(0.692-4.665) | 0.229 |  |  |
| Tumor histological grade* |  |  |  |  |
| Low | 1 |  |  |  |
| High | 1.742(0.613-4.946) | 0.297 |  |  |
| Lower edge of tumors with the dentate line invasion(yes vs. no) | 3.082(1.083-8.769) | 0.035 | 3.843(1.236-11.949) | 0.020 |
| c T Stage (4 vs. 2-3) | 2.055(0.759-5.568) | 0.157 |  |  |
| c N Stage (2 vs.0-1) | 0.815(0.186-3.568) | 0.786 |  |  |
| Induction chemotherapy (yes vs. no) | 0.042(0.000-48.814) | 0.380 |  |  |
| Concurrent chemotherapy |  |  |  |  |
| Capecitabine | 1 |  |  |  |
| CAPEOX | 1.405(0.458-4.312) | 0.552 |  |  |
| Consolidation chemotherapy |  |  |  |  |
| No | 1 |  |  |  |
| Capecitabine | 0.841(0.271-2.611) | 0.765 |  |  |
| CAPEOX | 0.820(0.107-6.313) | 0.849 |  |  |
| Preoperative serum CEA level (ng/mL) (>5 vs. ≤5) | 1.340(0.385-4.665) | 0.646 |  |  |
| Resection margin (R1 vs. R0) | 9.290(1.085-79.529) | 0.042 | 22.029(2.158-224.903) | 0.009 |
| yp T Stage(3-4 vs. 0-2) | 1.714(0.838-3.505) | 0.140 |  |  |
| yp N Stage(N+ vs. N0) | 2.567(0.835-7.885) | 0.100 |  |  |
| ypCR(yes vs. no) | 0.391(0.089-1.713) | 0.213 |  |  |
| Lymphovascular invasion(positive vs. negative) | - | - |  |  |
| Perineural invasion(positive vs. negative) | 2.538(0.574-11.218) | 0.219 |  |  |
| Adjuvant chemotherapy |  |  |  |  |
| No | 1 |  |  |  |
| Capecitabine | 2.324(0.655-8.246) | 0.192 |  |  |
| CAPEOX or mFOLFOX6 | 1.795(0.602-5.353) | 0.294 |  |  |
| Surgical technique **(**Laparoscopic vs. Open surgery) | 0.407(0.143-1.157) | 0.092 |  |  |
| Operation duration(min) | 0.996(0.989-1.004) | 0.346 |  |  |
| Intraoperative blood loss (ml) (>100 vs.≤100) | 2.145(0.791-5.814) | 0.134 |  |  |
| Number of lymph nodes examined in the surgery | 0.944(0.847-1.051) | 0.294 |  |  |
| Postoperative complications |  |  |  |  |
| No | 1 |  |  |  |
| Clavien- Dindo Grade I-II | 1.768(0.681-4.591) | 0.242 |  |  |
| Clavien- Dindo Grade III-V | - | 0.984 |  |  |

Abbreviations: CEA=carcinoembryonic antigen; c=clinical; DRFS= distant relapse-free survival; ECOG=Eastern Cooperative Oncology Group; pCR=pathologic complete response; RT=radiotherapy; yp=yield pathological;

*Evaluated by pretreatment diagnostic biopsy. Low indicates well or moderately differentiated; high indicates poorly differentiated, mucinous, or signet ring cell carcinoma.

| **Supplementary Table 5:** Univariate and multivariate Cox proportional hazards model for DFS after matching. | | | | | |
| --- | --- | --- | --- | --- | --- |
| Variable | Univariate | | | Multivariate | |
|  | HR (95% CI) | *P* | | HR (95% CI) | *P* |
| Sex (male vs. female) | 1.004(0.371-2.718) | | 0.993 |  |  |
| Age (y) (>60 vs. ≤60) | 1.542(0.608-3.909) | | 0.362 |  |  |
| ECOG (1 vs. 0) | 0.861(0.280-2.645) | | 0.793 |  |  |
| Baseline serum CEA level (ng/mL) (>5 vs. ≤5) | 1.961(0.775-4.962) | | 0.155 |  |  |
| Tumor histological grade* |  | |  |  |  |
| Low | 1 | |  |  |  |
| High | 1.742(0.613-4.946) | | 0.297 |  |  |
| Lower edge of tumors with the dentate line invasion(yes vs. no) | 3.023(1.063-8.601) | | 0.038 | 3.765(1.211-11.704) | 0.022 |
| c T Stage (4 vs. 2-3) | 1.719(0.665-4.440) | | 0.264 |  |  |
| c N Stage (2 vs.0-1) | 0.815(0.186-3.568) | | 0.786 |  |  |
| Induction chemotherapy (yes vs. no) | 0.042(0.000-48.814) | | 0.380 |  |  |
| Concurrent chemotherapy |  | |  |  |  |
| Capecitabine | 1 | |  |  |  |
| CAPEOX | 1.390(0.453-4.266) | | 0.564 |  |  |
| Consolidation chemotherapy |  | |  |  |  |
| No | 1 | |  |  |  |
| Capecitabine | 0.842(0.271-2.613) | | 0.766 |  |  |
| CAPEOX | 0.908(0.118-6.988) | | 0.926 |  |  |
| Preoperative serum CEA level (ng/mL) (>5 vs. ≤5) | 1.323(0.380-4.607) | | 0.660 |  |  |
| Resection margin (R1 vs. R0) | 9.290(1.085-79.529) | | 0.042 | 21.679(2.127-220.984) | 0.009 |
| yp T Stage(3-4 vs. 0-2) | 1.717(0.840-3.508) | | 0.138 |  |  |
| yp N Stage(N+ vs. N0) | 2.749(0.893-8.464) | | 0.078 |  |  |
| ypCR(yes vs. no) | 0.388(0.089-1.698) | | 0.209 |  |  |
| Lymphovascular invasion(positive vs. negative) | - | | - |  |  |
| Perineural invasion(positive vs. negative) | 2.931(0.658-13.049) | | 0.158 |  |  |
| Adjuvant chemotherapy |  | |  |  |  |
| No | 1 | |  |  |  |
| Capecitabine | 2.293(0.646-8.132) | | 0.199 |  |  |
| CAPEOX or mFOLFOX6 | 1.790(0.600-5.336) | | 0.296 |  |  |
| Surgical technique **(**Laparoscopic vs. Open surgery) | 0.412(0.145-1.171) | | 0.096 |  |  |
| Operation duration(min) | 0.996(0.989-1.003) | | 0.295 |  |  |
| Intraoperative blood loss (ml) (>100 vs.≤100) | 1.808(0.668-4.888) | | 0.244 |  |  |
| Number of lymph nodes examined in the surgery | 0.948(0.851-1.055) | | 0.325 |  |  |
| Postoperative complications |  | |  |  |  |
| No |  | |  |  |  |
| Clavien- Dindo Grade I-II | 1.945(0.769-4.923) | | 0.160 |  |  |
| Clavien- Dindo Grade III-V | - | | 0.984 |  |  |

Abbreviations: CEA=carcinoembryonic antigen; c=clinical; DFS=disease-free survival; ECOG=Eastern Cooperative Oncology Group; pCR=pathologic complete response; RT=radiotherapy; yp=yield pathological;

*Evaluated by pretreatment diagnostic biopsy. Low indicates well or moderately differentiated; high indicates poorly differentiated, mucinous, or signet ring cell carcinoma.
